# Aryl hydrocarbon receptor–kynurenine axis promotes oncogenic activity in BCP-ALL

**DOI:** 10.1007/s10565-022-09734-0

**Published:** 2022-06-10

**Authors:** Li-Ting Wang, Kwei-Yan Liu, Shen-Nien Wang, Ming-Hong Lin, Yu-Mei Liao, Pei-Chin Lin, Shau-Ku Huang, Shih-Hsien Hsu, Shyh-Shin Chiou

**Affiliations:** 1 Department of Life Science, National Taiwan Normal University, Taipei, Taiwan; 2 Department of Respirology & Allergy, Third Affiliated Hospital of Shenzhen University, Shenzhen, China; 3 Division of General and Digestive Surgery, Department of Surgery, Kaohsiung Medical University Hospital, Kaohsiung, Taiwan; 4 Graduate Institute of Medicine, College of Medicine, Kaohsiung Medical University, Kaohsiung, Taiwan; 5 Department of Surgery, College of Medicine, Kaohsiung Medical University Hospital, Kaohsiung, Taiwan; 6 Department of Microbiology and Immunology, School of Medicine, College of Medicine, Kaohsiung Medical University, Kaohsiung, Taiwan; 7 Department of Medical Research, Kaohsiung Medical University Hospital, Kaohsiung Medical University, Kaohsiung, Taiwan; 8 Division of Hematology-Oncology, Department of Pediatrics, Kaohsiung Medical University Hospital, Kaohsiung Medical University, Kaohsiung, Taiwan; 9 Department of Pediatrics, School of Post-Baccalaureate Medicine, College of Medicine, Kaohsiung Medical University, Kaohsiung, Taiwan; 10 National Institute of Environmental Health Sciences, National Health Research Institutes, Miaoli County, Taiwan; 11 Department of Medicine, Division of Allergy and Clinical Immunology, Johns Hopkins University School of Medicine, Baltimore, MD USA; 12 Research Center for Environmental Medicine, Kaohsiung Medical University, Kaohsiung, Taiwan; 13 Center of Applied Genomics, Kaohsiung Medical University, Kaohsiung City, Taiwan; 14 Graduate Institute of Clinical Medicine, College of Medicine, Kaohsiung Medical University, Kaohsiung, Taiwan

**Keywords:** AHR, BCP-ALL, PAH, KYN, IDO

## Abstract

B-cell precursor acute lymphoblastic leukemia (BCP-ALL), the most common childhood cancer, originates from lymphoid precursor cells in bone marrow committed to the B-cell lineage. Environmental factors and genetic abnormalities disturb the normal maturation of these precursor cells, promoting the formation of leukemia cells and suppressing normal hematopoiesis. The underlying mechanisms of progression are unclear, but BCP-ALL incidence seems to be increasing in parallel with the adoption of modern lifestyles. This study hypothesized that air pollution and haze are risk factors for BCP-ALL progression. The current study revealed that indeno(1,2,3-cd)pyrene (IP), a major component of polycyclic aromatic hydrocarbons (PAHs) in air, promotes oncogenic activities (proliferation, transformation, and disease relapse) in vitro and in vivo. Mechanistically, IP treatment activated the aryl hydrocarbon receptor (AHR)–indoleamine-2,3-dioxygenase (IDOs) axis, thereby enhancing tryptophan metabolism and kynurenine (KYN) level and consequent promoting the KYN–AHR feedback loop. IP treatment decreased the time to disease relapse and increased the BCP-ALL cell count in an orthotopic xenograft mouse model. Additionally, in 50 clinical BCP-ALL samples, AHR and IDO were co-expressed in a disease-specific manner at mRNA and protein levels, while their mRNA levels showed a significant correlation with disease-free survival duration. These results indicated that PAH/IP exposure promotes BCP-ALL disease progression.

**Graphical abstract:**

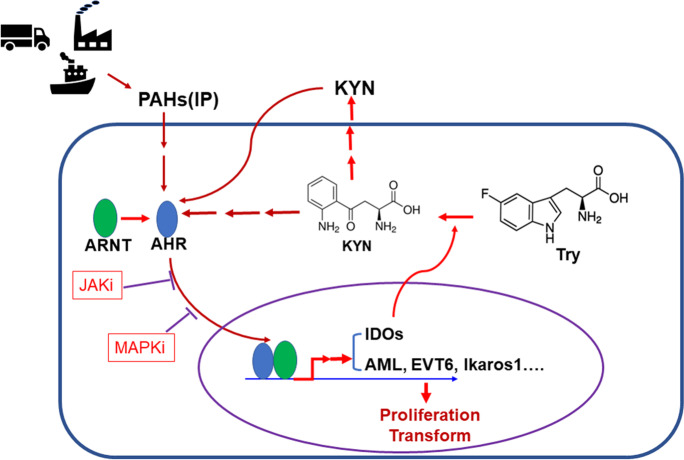

**Supplementary Information:**

The online version contains supplementary material available at 10.1007/s10565-022-09734-0.

## Introduction

Leukemia in children is mainly caused by the malignant proliferation of white blood cells(Lilljebjorn and Fioretos 2017). Although the cure rate for childhood acute lymphoblastic leukemia (ALL) has gradually increased in recent years, ALL relapse remains one of the most significant causes of childhood cancer mortality(Burgler and Nadal 2017; Jones et al. 2016). The risk factors for ALL initiation or relapse are correlated with exposure to exogenous and endogenous environments or genetic variation(Iacobucci and Mullighan 2017; Ghazavi et al. 2015; Tsai et al. 2015). Many exogenous environmental factors are considered significant risk factors, such as viral infection, radiation, environmental toxins (formaldehyde), and pollutants, including polycyclic aromatic hydrocarbons [PAHs])(Moriyama et al. 2015; Rodriguez-Villamizar, et al. 2020; Dehghani et al. 2017).

As primary organic fossil fuel (e.g., oil, coal) products, PAHs are critical environmental pollutants that endanger human health. Although some PAHs are considered carcinogens (e.g., benzo[*a*]pyrene)(Paris et al. 2018; Goudarzi et al. 2018), the cellular impact of chronic exposure to most PAHs is still unclear. Several studies have examined the effects of PAHs on carcinogenesis and tumor growth(Famiyeh et al. 2021). However, studies on PAHs in children with ALL are relatively rare, except chronic myelogenous leukemia. The relationship between PAH exposure and children’s carcinogenesis is unclear for ALL (78%) and B-cell precursor cell (BCP)-ALL (68%), which account for most cases of leukemia(Chen et al. 2015; An, et al. 2017). Leukemia epidemiological studies have not measured PAH concentrations in environmental media, and few such reports exist wordwide(Kislov et al. 2013; Marcoux et al. 2000). However, some studies found that PAH exposure, including parental occupational exposure to car exhaust, smoking, and proximity to transportation routes, is related to the risk of childhood leukemia(Weng et al. 2008). Although the research results are inconsistent, the relevant results suggested that PAH exposure increases the risk of ALL and affects the disease process(Chen et al. 2021a; Zhang et al. 2021a; Enuneku et al. 2021). Moreover, its regulatory mechanism appears related to the aryl hydrocarbon receptor (AHR) (O'Driscoll et al. 2018; Tao et al. 2021).

AHR is a basic cytosolic helix–loop–helix/Per–Arnt–Sim transcriptional factor that resides in the cytoplasm in its resting state(Nguyen et al. 2013). After ligand binding, AHR translocates to the nucleus, where it heterodimerizes with its binding partner AHR nuclear translocator and activates the expression of a battery of genes containing specific DNA target sequences (xenobiotic-responsive elements)(Puga et al. 2009). AHR activation functionally increases xeno-toxin metabolism, but recently it seems to increase cellular proliferation, anti-apoptotic activity, and angiogenesis in several cancer cell lines and animal models(Nebert et al. 2004). However, its mechanism is not completely defined. Studies indicated that AHR dysregulation has a vital role in hematopoietic cell progression toward malignancy(Dietrich and Kaina 2010).

Moreover, increased AHR expression has been noted in ALL, suggesting its potential pro-tumorigenic activity(Alicandro et al. 2016; Deziel et al. 2014; Metayer et al. 2016). However, the regulatory mechanisms of AHR expression and the functional impact on ALL have yet to be elucidated. Additionally, indoleamine-2,3-dioxygenase (IDO), a potential target of AHR(Zelante et al. 2014), facilitates tryptophan catabolism at the rate-limiting step of the kynurenine (KYN) pathway(Wang et al. 2017; Wu et al. 2021). IDO expression and increased KYN metabolite levels are associated with the immunosuppressive tumor microenvironment(Wu et al. 2021; Chen et al. 2021b; Zhang et al. 2021b). Signaling in immune cell types through KYN and its receptor AHR increases the apoptosis of T-helper 1 lymphocytes and natural killer cells, thereby preventing excessive immune activity(Chen et al. 2021b; Li et al. 2020). This study presents comprehensive evidence that AHR activity induced by indeno(1,2,3-cd)pyrene (IP) through AHR–IDO–KYN signaling promotes oncogenesis in BCP-ALL.

## Materials and methods

### Plasmids, cell lines, and other materials

The *PLKO.1.puro* or*.neo* vector was used as a backbone for *shRNAi* constructs targeting *AHR.* The shRNAi sequence of *AHR* was showed as follows: *AHR* shRNAi 5′-CGGCATAGAGACCGACTTAAT-3′. Transfection was performed using the LipofectAMINE transfection kit (Gibco, Thermo Fisher Scientific, Waltham, MA, USA). Human leukemia cell lines (Molt-4, REH, Jurkat, Sup-B15, Nalm-6, B-job, HL60, and K562) and lymphoblastoid cell lines (LCLs) were obtained from ATCC and maintained according to the manufacturer’s protocol. Indeno(1,2,3-cd)pyrene (ERI-001) was purchased from Sigma-Aldrich (Saint Louis, MO, USA) and dissolved in methanol. Chemical and kinase inhibitors were purchased from Sigma-Aldrich (Saint Louis, MO, USA) and AKT (124,005), p38 (S8307), U0126 (19–147), JAK (420,099), PI3K (L9908), NF-kB (B5556), INCB, and KYN (K8625). IDO inhibitors (INCB024360 and NLG8189) were purchased from MyBioSource.

### Western blotting and immunohistochemical analysis

Western blotting and immunohistochemical (fluorescence) staining were performed as described previously (Hsu et al. 2013; Chiou et al. 2014). The primary antibodies used in this study were Cyclin D1 (1:200), and actin polyclonal antibodies (1:5000 dilution; Sigma-Aldrich). The AHR and Ki67 goat polyclonal antibody (1:200 dilution; Santa Cruz Biotechnology). The Ikaros1, E2F1, IDO1, ETV6, TDO2, IDO2, CYP1B1, AML1, OCT4, and Ikaros1 (1:500 dilution) primary antibody was obtained from GeneTex International Corp. All of the experiments were repeated at least 3 times.

### RNA isolation

In order to compare the relative gene expression profiles in ALL patients, total RNA from liver tissue RNA were isolated by RNeasy Mini Kit according to instructions from the manufacturer (Qiagen, Valencia, CA, USA).

### Real-time PCR

The expressions of *IDO1*, *TDO2*, and *AHR* mRNA in leukemia cells from cancer patients were quantified using the SYBR Green Quantitative RT-PCR kit (Invitrogen) as described previously. The total RNA was extracted from the tumor mass using TRIzol reagent (Invitrogen) and then transcribed into cDNA (Invitrogen) for PCR amplification on a 7900HT Thermocycler (Applied Biosystems Inc.). All procedures and data analysis were performed according to the manufacturer’s instructions. All data are expressed as the mean ± SD of at least 3 experiments.

### Cell proliferation assay

A colorimetric immunoassay (Roche) for the quantification of the cell proliferation was performed based on the measurement of BrdU incorporation during DNA synthesis according to the manufacturer’s instructions. The cells (10^4^) cultured in 96-well plates were incubated at 37 °C for 16 h and then growth-arrested prior to the indicated treatment. The BrdU labeling reagent was added into the medium for a 2-h incubation at 37 °C. Absorbance values were measured at 495 nm using a VersaMax ELISA Microplate Reader.

### Anchorage-independent growth assays

Cells (10^4^ or 5 × 10^3^) in 1 mL of a culture medium were mixed with an equal volume of 0.6% top agar and plated onto 60-mm culture dishes with 0.5% bottom agar. The plates were incubated at 37 °C for 2 weeks. Colonies were visualized by staining with 0.05% crystal violet acetate, and only those larger than 0.5 mm were counted. The culture medium was replaced every 3 days. All data are expressed as the mean ± SD of at least 3 experiments.

### ELISA that measures human kynurenine concentrations in culture medium

This was conducted using the human Kynurenine ELISA Kit from MyBioSource, San Diego (USA) (MBS704658), according to the manufacturer’s instructions. Human BCP-ALL cells treated with IP or inhibitor seeded in 96-well culture plates were incubated at 37 °C. After 24 h, the conditioned medium was collected and centrifuged at 3000 rpm for 20 min to remove cell debris. The supernatants were then subjected to ELISA in triplicates. Kynurenine concentrations were computed with reference to standard curves derived from purified kynurenine supplied in the ELISA Kit.

### Patients

This study enrolled 50 ALL patients (27 men and 23 women; mean age, 9.1 ± 5.83 years; range, 0.5–24 years) from three medical centers (Chung Ho Memorial Hospital (50 ALL)) were enrolled to the AHR, TDO2, and IDO1 cohort study from May 2016 to March 2022. The study was conducted with approval (KMUH-IRB-20160025 and 20,210,034) from the ethics committee of Kaohsiung Medical University Chung-Ho Memorial Hospital. Written informed consent was obtained from each patient.

### Leukemia mice model

We used 8-week-old male non-obese diabetic/severe combined immunodeficiency (NOD-SCID) mice obtained from the National Laboratory of Animal Breeding and Research Center (Taipei, Taiwan). Nalm-6 cells (5 × 10^7^) were injected into non-obese diabetic/severe combined immunodeficiency (NOD-SCID) mice via the tail vein injection to evaluate the oncogenic activity of IP. After 1 week, the mice were treated with IP (40 ng/g 3 days) for 50 days. All animal experiments were approved by the Kaohsiung Medical University–Institutional Animal Care and Use Committee (IACUC-108211; Kaohsiung, Taiwan) and were in accordance with the guidelines (KMU Animal Care and Use Program for Center for Laboratory Animals, Kaohsiung Medical University, Taiwan) and regulations of the institution. Male NOD-SCID mice were obtained from the National Laboratory of Animal Breeding and Research Center (Taipei, Taiwan). NOD-SCID mice were inoculated (s.c. injection) with 10^7^ Nalm-6 cells individually (*n* = 10 mice/group).

### Statistical analysis

Quantitative variables are presented as mean ± SD. The significance of differences was determined using a two-sample *t*-test and non-parametric analysis. Statistical analysis of categorical variables was performed using chi-squared analysis, one-way analysis of variance, and Fisher’s exact analysis. Differences with a *P* value < 0.05 were considered significant.

## Results

### BCP-ALL cells treated with IP display significantly increased cell proliferation and higher stem cell-like and lipid metabolism marker levels

**Fig. 1:**
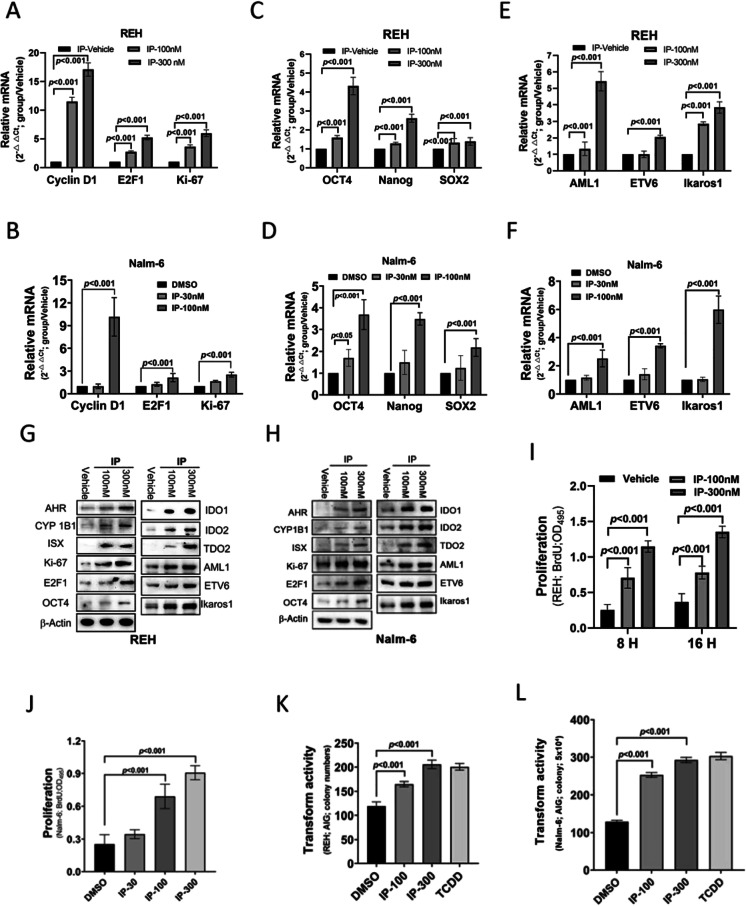
B-cell precursor acute lymphoblastic leukemia cells treated with indeno(1,2,3-cd)pyrene (IP) exhibited increased expression of cell proliferation, stem cell-like, and lipid metabolism markers. **A**, **B** The mRNA expression levels of Cyclin D1, E2F1, and Ki-67 relative to GAPDH in REH and Nalm-6 cells treated with IP as detected by semiquantitative reverse transcription-polymerase chain reaction (RT-PCR). Data are presented as the mean ± SD. *a*, *P* < 0.001. **C**, **D** The mRNA expression levels of OCT4, Nanog, and SOX2 relative to GAPDH in REH and Nalm-6 cells treated with IP as detected by semiquantitative RT-PCR. Data are presented as the mean ± SD. *a*, *P* < 0.001. **E**, **F** The mRNA expression levels of AML1, ETV6, and Ikaros1 relative to GAPDH in REH and Nalm-6 cells treated with IP as detected by semiquantitative RT-PCR. Data are presented as mean ± SD. *a*, *P* < 0.001. **G**, **H** The protein expression of cell proliferation, stem cell-like, and lipid metabolism markers in REH and Nalm-6 cells treated with IP was detected by western blotting. **I**, **J** Proliferative activity of REH and Nalm-6 cells treated with IP as measured by bromodeoxyuridine incorporation. **K** and **L** Analysis of the transformation of REH and Nalm-6 cells treated with IP or 2,3,7,8-tetrachlorodibenzo-*p*-dioxin (TCDD), and cell anchorage-dependent transformation was measured using the soft agar colony formation assay. *a*, *P* < 0.001, comparing different experimental conditions as indicated

First, we analyzed the expression patterns of proliferation-related genes (cyclin D1, E2F1, and Ki-67), stem cell-like (OCT4, Nanog, and SOX2), and leukemia-associated mutant markers (AML1, ETV6, and Ikaros1) in BCP-ALL cells (REH and Nalm-6) treated with IP to explore the potential functional relationship between IP and BCP-ALL. Cell proliferation marker expression increased in BCP-ALL cells treated with IP in a concentration-dependent manner (Fig. 1A and B). OCT4, Nanog, and SOX2 expression was significantly increased in BCP-ALL cells treated with IP (Fig. 1C and D), as was AML1, ETV6, and Ikaros1 mRNA expression (Fig. 1E and F).

### IP promotes BCP-ALL cell proliferation and transformation

We evaluated the proliferation of IP-responsive cells and expression of stem cell-like and leukemia-associated mutant markers in BCP-ALL cells treated with IP to assess potential tumorigenesis. Similar to those observed at the mRNA level, the protein expression of cell proliferation, stem cell-like, and leukemia-associated mutant markers was increased in BCP-ALL cells treated with IP (Fig. 1G and H). The cellular and oncogenic activities regulated by IP were further examined regarding their effects on cell proliferation and transformation in vitro. Firstly, BCP-ALL cells treated with IP displayed increased proliferation in the bromodeoxyuridine (BrdU) incorporation assay (Fig. 1I and J). Secondly, cellular transformation in BCP-ALL cells was enhanced by IP in an independent soft agar assay, in which treatment with IP and 2,3,7,8-tetrachlorodibenzo-*p*-dioxin (TCDD), a well-established AHR ligand for comparison, significantly increased colony formation (Fig. 1K and L).

### IP induces AHR expression, cell proliferation, and cell transformation through the JAK and PI3K signaling pathways

**Fig. 2:**
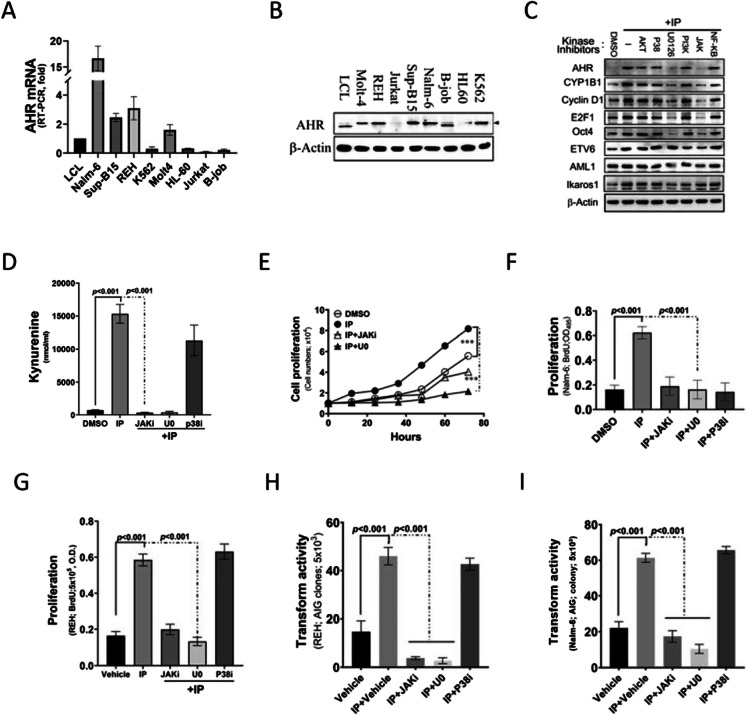
Indeno(1,2,3-cd)pyrene (IP)-induced aryl hydrocarbon receptor (AHR) expression, cell proliferation, and cell transformation through the JAK and PI3K signaling pathways. **A** Endogenous mRNA expression of AHR in various leukemia cell lines as detected by semiquantitative reverse transcription-polymerase chain reaction. **B** Endogenous protein expression of AHR in various leukemia cell lines as detected by western blotting. **C** Western blotting of AHR, CYP1B1, CD86, PD-L1, E2F1, OCT4, ETV6, AML1, and Ikaros1 protein expression in REH cells treated with IP and kinase inhibitors. **D** Kynurenine (KYN) levels in REH cells treated with IP, JAK and p38 kinase inhibitors, and DMSO (control). *a*, *P* < 0.001. **E**–**G** Proliferative activity of REH and Nalm-6 cells treated with IP and kinase inhibitors as measured by cell counting and bromodeoxyuridine incorporation assays. **H**, **I** Analysis of the transformation of REH and Nalm-6 cells treated with IP and kinase inhibitors. Cell anchorage-dependent transformation was measured using the soft agar colony formation assay. *a*, *P* < 0.001, comparing different experimental conditions as indicated

We analyzed AHR expression patterns in leukemia cell lines to explore the potential functional relationship among IP, AHR, and BCP-ALL. Analyses of eight leukemia cell lines (Nalm-6, Sup-B15, REH, Molt-4, K562, HL-60, Jurkat, and B-job) revealed higher AHR expression in B-cell leukemia cells (Nalm-6, REH, and Sup-B15) than in lymphoblastoid cell lines (Fig. 2A and B). Furthermore, REH cells treated with IP were separately co-incubated with different kinase-specific inhibitors to evaluate transcriptional activation. Moreover, we analyzed the expression of AHR and cell proliferation markers via western blotting. The protein expression of AHR and cell proliferation markers induced by IP treatment was concentration-dependently suppressed by the JAK-specific inhibitor AG480 and MAPK-specific inhibitor U0126 (Fig. 2C). Meanwhile, IP increased KYN secretion in REH cells, and inhibition of JAK and MAPK in REH cell lines reversed this effect (Fig. 2D). Moreover, we examined the functional effects of IP on BCP-ALL cells. The results indicated that IP significantly increased the cell growth (Fig. 2E), proliferation (Fig. 2F and G), and transformation of BCL-ALL cells (Fig. 2H and I), and these effects were reversed by treatment of the cells with AG480 and U0126.

### AHR expression is essential for proliferation and transformation in leukemia cells treated with IP

**Fig. 3:**
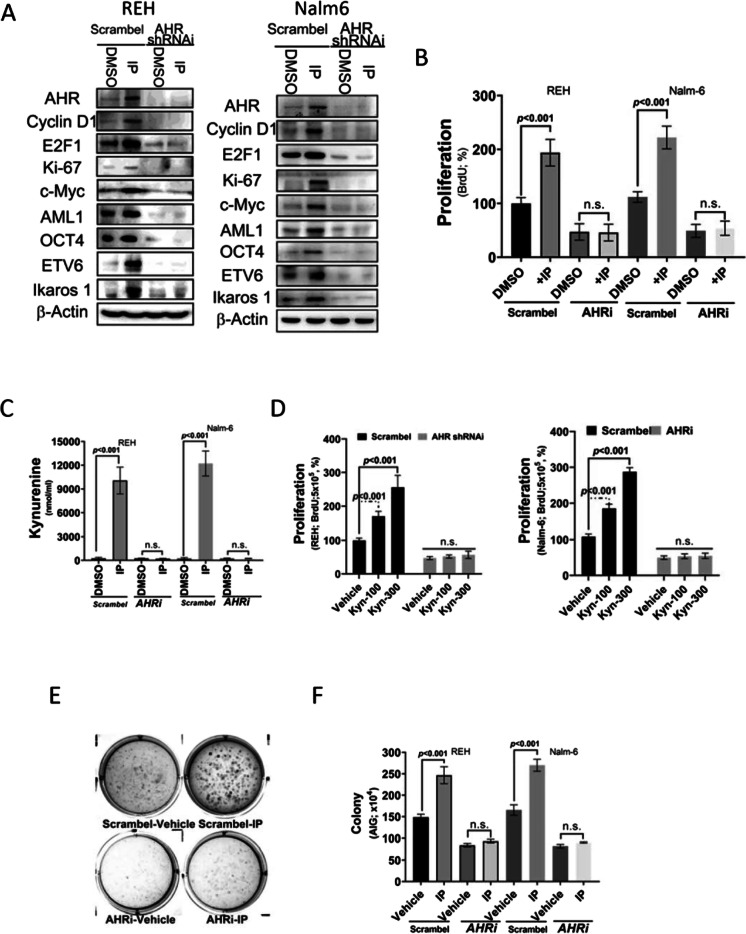
Aryl hydrocarbon receptor (AHR) expression-induced proliferation and transformation in leukemia cells. **A** Western blotting of AHR, cyclin D1, E2F1, c-Myc, Ki-67, OCT4, ETV6, Ikaros1, and AML1 protein expression in indeno(1,2,3-cd)pyrene (IP)-stimulated REH and Nalm-6 cells transfected with AHR short hairpin RNA (shRNA), vehicle, or vector (pLKO). **B** Proliferation of REH and Nalm-6 cells following AHR shRNA or pLKO transfection and treated with IP as measured by bromodeoxyuridine incorporation. **C** KYN levels in IP-stimulated REH and Nalm-6 cells following AHR shRNA or pLKO transfection. **D** Proliferation of REH and Nalm-6 cells treated with KYN or vehicle. *a*, *P* < 0.001, comparing different experimental conditions as indicated. **E**, **F** Analysis of the transformation of IP-stimulated REH and Nalm-6 cells following AHR shRNA or pLKO transfection, and cell anchorage-dependent transformation was measured using the soft agar colony formation. *a*, *P* < 0.001, comparing different experimental conditions as indicated

Endogenous AHR was silenced in REH and Nalm-6 cells using four short hairpin RNA interference to determine whether AHR is essential for IP-induced signaling (Figure S1). AHR-silenced cells were then treated with IP to evaluate the regulatory effects of AHR on cell proliferation. IP activated AHR and cell proliferation marker (Cyclin D1, E2F1, and Ki-67) expression at the protein level (Fig. 3A). Also, IP activated BCL-ALL oncogenic marker (AML1, c-Myc, ETV6, and Ikarose1) expression at the protein level (Fig. 3A) However, IP-induced AHR, cell proliferation, and oncogenic marker activation of BCL-ALL was abrogated in AHR-silenced cells compared to the findings in mock-infected cells (Fig. 3A). The proliferation and transformation of AHR-silenced leukemia cells were determined using BrdU incorporation and independent soft agar assays to further assess the tumorigenic effect of AHR on leukemia prognosis. IP-induced AHR expression increased BrdU incorporation in REH and Nalm-6 cells, but AHR-silenced cells exhibited decreased proliferation and transformation (Fig. 3B, E, and F) even at higher doses of IP exposure. IP increased KYN secretion in REH and Nalm-6 cells and silencing of AHR in REH and Nalm-6 cell lines reversed this effect (Fig. 3C). KYN increased cell proliferation in REH and Nalm-6 cells, but AHR-silenced cells exhibited decreased proliferation (Fig. 3D).

### IDO inhibitors significantly inhibit AHR-mediated KYN expression, cell proliferation, and cell transformation

**Fig. 4:**
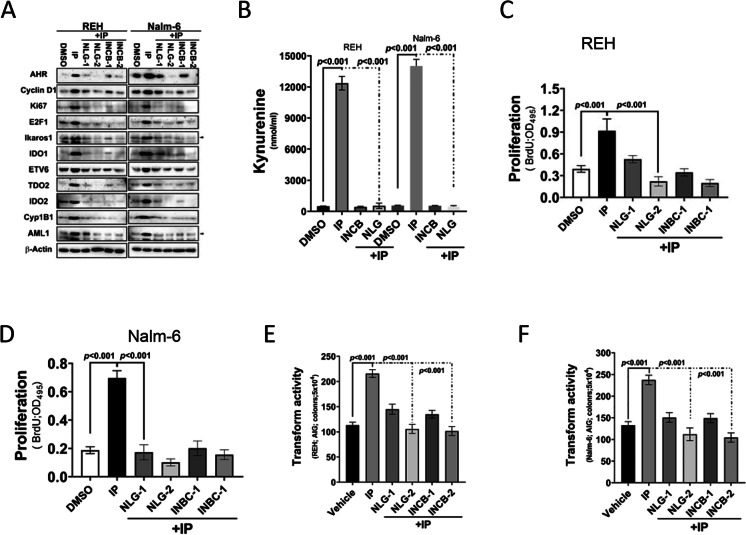
Indoleamine-2,3-dioxygenase (IDO) inhibitors suppressed aryl hydrocarbon receptor (AHR)-mediated increases of kynurenine (KYN) release, cell proliferation, and cell transformation. **A** Western blotting of AHR, cyclin D1, Ki-67, E2F1, Ikaros1, IDO1, ETV6, TDO2, IDO2, CYP1B1, and AML1 protein expression in REH and Nalm-6 cells treated with indeno(1,2,3-cd)pyrene (IP) and the IDO inhibitors INCB 024,360 and NLG-8189. **B** KYN levels in REH cells treated with IP, INCB 024,360, and NLG-8189. *a*, *P* < 0.001. **C**, **D** Proliferation of REH and Nalm-6 cells treated with IP, INCB 024,360, and NLG-8189 as measured by bromodeoxyuridine incorporation. **E**, **F** Analysis of the transformation of REH and Sup-B15 cells treated with IP, INCB 024,360, and NLG-8189, and cell anchorage-dependent transformation was measured using the soft agar colony formation. *a*, *P* < 0.001, comparing different experimental conditions as indicated

IDOs control tryptophan catabolism (9), whereas IP influences KYN–AHR oncogenic signaling (12). We measured KYN expression and oncogenic activity in leukemia cells (REH and Nalm-6) to verify the regulatory effects of IP on AHR–IDO–KYN signaling. We induced Nalm-6 cells to express AHR via IP treatment in the presence of IDO-selective inhibitors (INCB024360, 5 and 10 mmol/L; NLG-8189, 15 and 30 mmol/L). Both IDO inhibitors significantly suppressed, albeit at varying degrees, AHR, IDO1, IDO2, TDO2, Ikaros1, ETV6 AML1, and cell proliferation marker (Cyclin D1, E2F1, and Ki-67) expression (Fig. 4A). KYN levels were increased in leukemia cells treated with IP, thereby inducing AHR expression. However, the IP-mediated upregulation of its targets and KYN release were inhibited in leukemia cells treated with IP and IDO inhibitors (Fig. 4B). Furthermore, IP-induced AHR expression increased BrdU incorporation and transformation activity in B-cell leukemia cells (REH and Nalm-6), but cells treated with IDO inhibitors exhibited decreased proliferation and transformation (Fig. 4C–F).

### KYN promotes leukemia cell proliferation and transformation

**Fig. 5:**
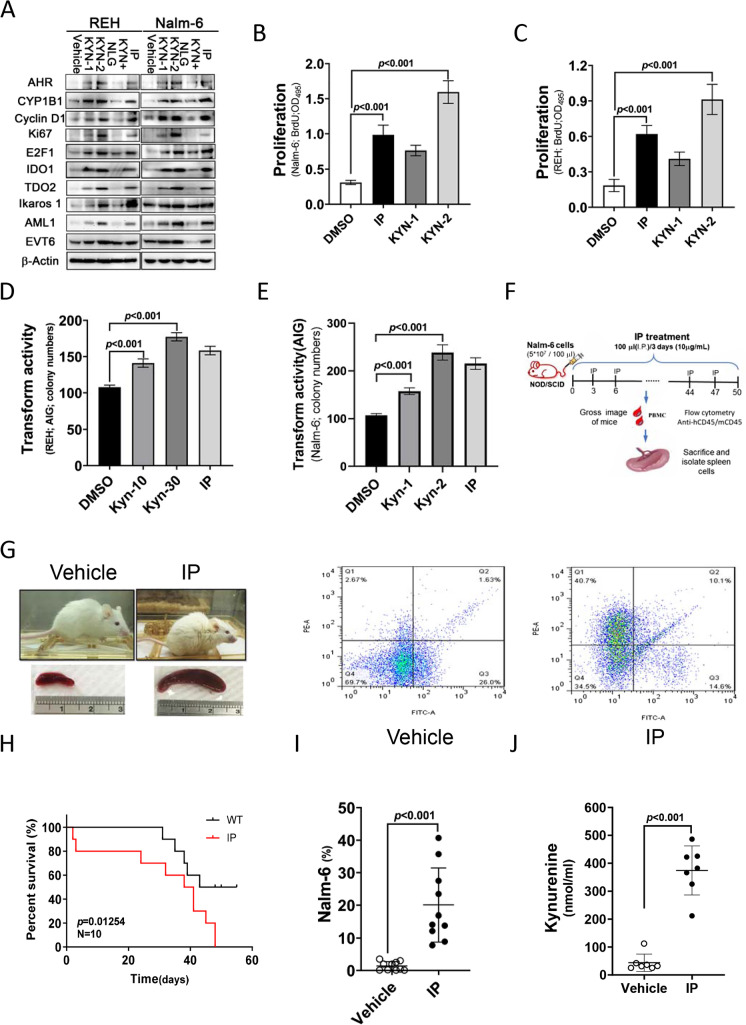
Kynurenine (KYN) promoted the proliferation and transformation of leukemia cells. **A** Western blotting of aryl hydrocarbon receptor (AHR), CYP1B1, cyclin D1, Ki-67, E2F1, IDO1, TDO2, Ikaros1, AML1, IDO2, and ETV6 protein expression in REH and Nalm-6 cells treated with IP plus KYN or vehicle in the presence or absence of an indoleamine-2,3-dioxygenase inhibitor. **B**, **C** Proliferation of REH and Nalm-6 cells treated with IP and KYN as measured by bromodeoxyuridine incorporation. **D**, **E** Analysis of the transformation of REH and Nalm-6 cells treated with IP and KYN, and cell anchorage-dependent transformation activity was determined using the soft agar colony formation. *a*, *P* < 0.001, comparing different experimental conditions as indicated. **F** A timeline of the experiment conducted in the present **G** Spleen evaluation of an orthotopic xenograft mouse model established from Nalm-6 cells and treated with vehicle or indeno(1,2,3-cd)pyrene (IP). PE-hCD45; FITC-mCD45. **H** Survival curve analysis of NOD-SCID mice injected with Nalm-6 cells and treated with vehicle or IP. **I** IP treatment mice significantly increased the proportion of Nalm-6 cells in the mouse spleen compared to the effects of vehicle. **J** KYN levels in NOD-SCID mouse serum

We then examined the functional effects of KYN and its positive feedback loop on leukemia cells. The results indicated that KYN significantly increased cell proliferation marker expression (cyclin D1, E2F1, and Ki-67), as well as IDO1, TDO2, Ikaros1, AML1, and ETV6 expression, in leukemia cells (Fig. 5A). Meanwhile, IDO inhibition decreased their expression. Similarly, leukemia cell proliferation and transformation following KYN treatment were examined using BrdU incorporation and independent soft agar assays. The results illustrated that KYN increased leukemia cell proliferation and transformation (Fig. 5B–E).

### IP significantly induces Nalm-6 cell proliferation in an orthotopic xenograft mouse model

Nalm-6 cells were injected into non-obese diabetic/severe combined immunodeficiency (NOD-SCID) mice via the tail vein injection to evaluate the oncogenic activity of IP. After 1 week, the mice were treated with IP (40 ng/g 3 days) for 50 days (Fig. 5F). Spleen cells were stained with human and mouse CD45 monoclonal antibodies to reveal the onset of BCP-ALL, and human CD45 + cells were counted as cell oncogenic proliferation markers. IP treatment significantly increased Nalm-6 cell proliferation compared to the effects of vehicle treatment in NOD-SCID mice (Fig. 5G). IP-treated mice displayed shorter survival than vehicle-treated mice (Fig. 5H). In addition, IP-treated mice had a significantly higher percentage of Nalm-6 cells and higher serum KYN levels than vehicle-treated mice (Fig. 5I and J).

### AHR–IDO–KYN axis expression is significantly correlated with the clinical outcome of patients with BCP-ALL

To evaluate the clinical potential outcome of AHR, 50 patients with BCP-ALL from the Kaohsiung Medical University Hospital were enrolled in the AHR cohort study between August 2006 and November 2020. Forty of the patients with BCP-ALL originally enrolled had sufficient follow-up data for analysis. All patients were treated by following the protocols of the Taiwan Pediatric Oncology Group. The baseline characteristics of the patients based on the categorization of AHR expression are shown in Table 1. The average follow-up time for all patients was 60 months. Bone marrow cells were harvested from the ALL patients at diagnosis or relapse and the normal controls were obtained after informed consent. Further, to classify the regulation effect on leukemia patients, the expression level of AHR in patients with ALL was defined as the high group, in which AHR mRNA detected was sixfold higher than that in a normal donor. There were significant differences between low- or high-level groups of AHR expression and disease relapse (*P* = 0.01) and disease-free survival (*P* < 0.01). In contrast, there was no correlation between AHR expression and age, gender, white blood cell count, hemoglobin levels, platelet count, and levels of hepatomegaly or splenomegaly (Table 1). This suggests that AHR expression has a significant regulatory effect on patient relapse and overall survival in patients with BCP-ALL.

**Table 1:** Demographic data of 50 patients with B-cell precursor acute lymphoblastic leukemia (BCP-ALL)

	Low AHR (*N* = 26)		High AHR (*N* = 24)		*P* value
Age					0.81
< 2 y	5	19.2%	3	12.5%	
≥ 2 y and < 10 y	11	42.3%	11	45.8%	
≥ 10 y	10	38.5%	10	41.7%	
Sex					0.79
Male	15	57.7%	12	50%	
Female	11	42.3%	12	50%	
WBC					0.56
≤ 50,000/μl	12	46.2%	14	58.3%	
> 50,000/μl	14	53.8%	10	41.7%	
Platelet					0.79
≤ 150 × 10^3^/μl	14	53.8%	13	54.2%	
> 150 × 10^3^/μl	12	46.3%	11	45.8%	
Hemoglobin					0.91
≤ 12 g/dl	17	65.4%	17	70.8%	
> 12 g/dl	9	34.6%	7	29.2%	
Hepatomegaly					0.81
Yes	15	57.7%	14	58.3%	
No	11	42.3%	10	41.7%	
Splenomegaly					0.27
Yes	9	34.6%	13	54.2%	
No	17	65.4%	11	45.8%	
Lymph node number					0.01**
≤ 2	18	69.2%	14	58.3%	
> 2	8	30.8%	10	41.7%	
CNS involvement					0.02*
Yes	6	23.1%	14	58.3%	
No	20	76.9%	10	41.7%	
Relapse					0.01**
Yes	8	30.8%	17	70.8%	
No	18	69.2%	7	29.2%	
Death					0.006**
Yes	2	7.7%	11	45.8%	
No	24	92.3%	13	54.2%	

The BCP-ALL patients were classified into two groups—the “low” group and “high” group, according to survival ROC curve analysis. The cutting points of AHR were 6.0 times of the average level in controls. The 2-sample chi-square test was used to compare the differences between BCP-ALL patients with low and high AHR mRNA

^*^p < 0.05; **p < 0.01

**Fig. 6:**
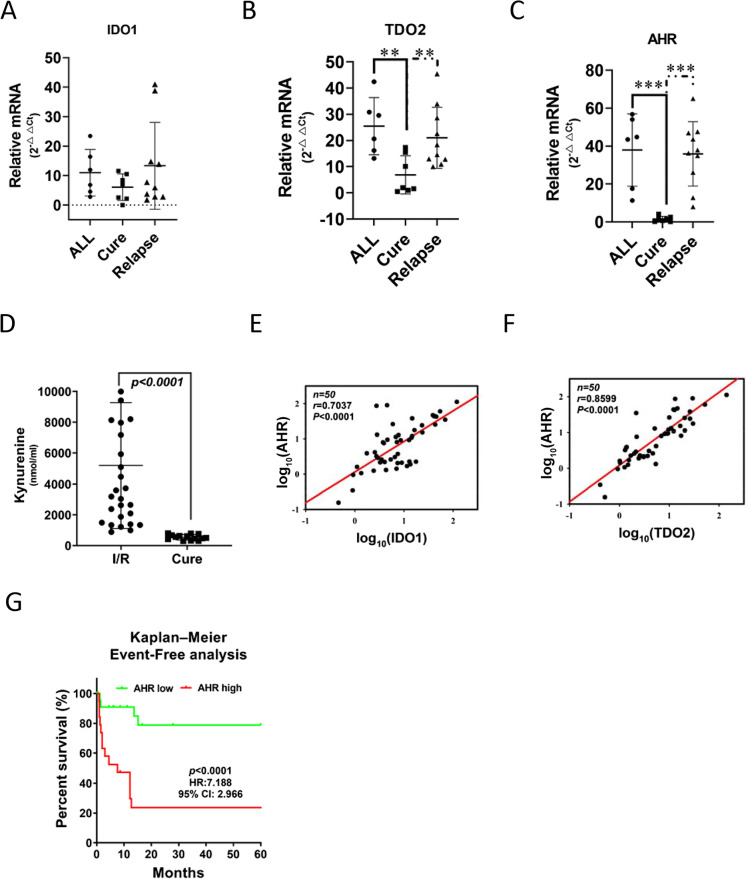
Aryl hydrocarbon receptor (AHR)–indoleamine-2,3-dioxygenase (IDO)–kynurenine (KYN) axis expression is correlated with the clinical outcomes of patients with B-cell precursor acute lymphoblastic leukemia (BCP-ALL). **A**–**C** Relative mRNA expression of IDO1, TDO2, and AHR in the blood of patients with various stages of BCP-ALL (primary, remission, relapse) as determined by semiquantitative reverse transcription-polymerase chain reaction. *a*, *P* < 0.001. **D** KYN levels in the blood of patients with primary, cured, and relapsed BCP-ALL were determined by enzyme-linked immunosorbent assay. **E**, **F** Correlation analysis of mRNA expression for, (E) AHR and IDO1, (F) AHR and TDO2 in 50 BCP-ALL samples. **G** The Kaplan–Meier event-free survival curve was used to analyze the event-free survival correlation between patients (*n* = 50) with BCP-ALL and AHR levels. On the basis of the cut-off values of fold differences, the study population was dichotomized into the “high” and “low” expression groups. *P* values were calculated by the log-rank (Mantel–Cox) test comparing the two Kaplan–Meier curves

Further, to characterize the potential correlation between prognosis and AHR–IDO–KYN signaling clinically, the mRNA expression of these genes and the level of KYN in 50 BCP-ALL samples were examined. IDO1, KYN, AHR, and TDO2 expression was correlated with clinical outcomes. Patients with primary or relapsed ALL exhibited significantly higher IDO1, AHR, TDO2, and KYN levels than patients without relapse (Fig. 6A–D). Moreover, mRNA expression of ISX strongly correlated with those of IDO1 and TDO2 in patients with BCP-ALL (Pearson’s correlation coefficient, *r* = 0.7037 and 0.8599, respectively, *P* < 0.0001; Fig. 6E and F). In the analyses of disease-free survival, patients with low AHR expression had a significantly longer survival time than those with high expression following treatment with established chemotherapy (Fig. 6G; *P* < 0.001; HR = 7.188).

## Discussion

**Fig. 7:**
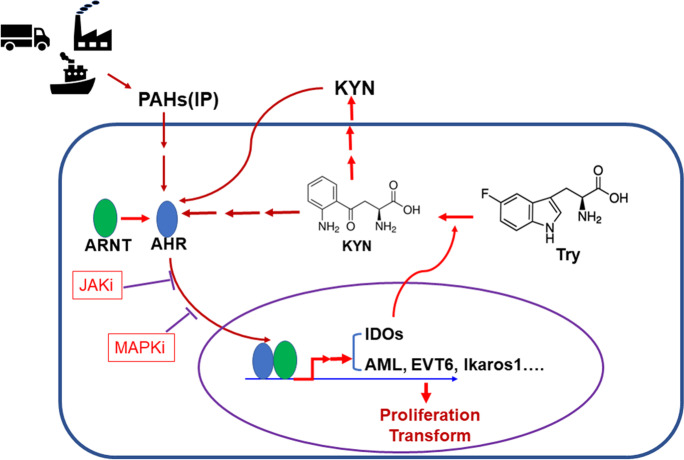
Working hypothesis of the mechanism that AHR–IDO–KYN signaling promotes IP-induced oncogenesis in BCP-ALL cells

Leukemia is the most common malignancy in children, and it primarily involves a multistep deregulatory genetic defect and environmental exposures. This study revealed that environmental pollutant, PAH(IP), exposure activates the AHR–IDOs axis basically and clinically. This activation enhances tryptophan metabolism and KYN release, further promoting KYN–AHR signaling and providing a potential linkage among environmental PAH exposure, tryptophan metabolism, and BCP-ALL development (Fig. 7).

IP is one of the five most common PAHs in air pollutants in many environmentally contaminated areas, such as industrial harbors or cities(Chen et al. 2021c; National Toxicology 2011); however, the cytotoxicity and disease impact after exposure are rare and unclear yet. More than 100 PAHs currently exist, including both natural and synthetic types. Most vapor PAHs have only two or three benzene rings; however, PAHs with more than five benzene rings form particles with a volume of < 2.5 μm(Russold et al. 2006) and are major haze as carcinogens in air pollutants (Sopian et al. 2021). Destroying the genes in the cell causes genetic mutations and promotes cancer cell growth(Longoria-Rodriguez et al. 2020). We analyzed proliferation marker expression in BCP-ALL cell lines to further clarify PAH-induced cell proliferation and tumorigenesis. The results illustrated that proliferation and transformation were significantly increased by IP treatment in BCP-ALL cells.

PAHs may indeed increase the risk of AML and affect the course of the disease(Baum et al. 2020). The related regulatory mechanism appeared to be related to AHR, a unique chemical sensor activated by xenobiotics and a major regulator of xenobiotic-induced carcinogenesis, including leukemogenesis(Yang et al. 2021). However, the role of AHR in the processes of tumor initiation and development remains to be elucidated. This study found that IP-induced AHR expression and AHR-relevant oncogenic effects on proliferation and transformation in BCP-ALL cells. Contrarily, IP-induced oncogenic effects on proliferation or transformation signaling were abrogated in AHR-silenced BCP-ALL cells. These results clearly revealed that the IP–AHR axis promotes the development of BCP-ALL.

Kynurenine (KYN) is an important bioactive metabolite of tryptophan metabolism via tryptophan dioxygenase (Platten et al. 2012), such as indoleamine 2,3-dioxygenase (IDO) (Jasperson et al. 2009). IDOs, enzymes that catalyze tryptophan degradation, were majorly regulated through JAK/STAT signaling(Arumuggam et al. 2015). Also, MAPK signals activated the IDO1 level via the kynurenine/AhR perturbation loop pathway in gastric cancer(Xiang et al. 2019). Recently, evidences showed kynurenine carried out a diversity of biological functions, including dilating blood vessels during inflammation (Liu et al. 2019), regulating the immune response (Wang et al. 2010), and increasing solid tumor growth (Platten et al. 2012). Many studies also showed that increased kynurenine levels were detected and may precipitate disease development, such as cognitive deficits in schizophrenia (Schwarcz et al. 2012), Alzheimer’s disease (Dounay et al. 2015), and cardiovascular disease(Schwarcz et al. 2012). This study also noted a parallel and interactive event in which the AHR–KYN axis upregulated proliferation and transformation through the effects of IP in children BCP-ALL patients. The findings in REH and Nalm-6 cells treated with KYN and IDO inhibitors confirmed the role of the AHR–IDO–KYN axis in leukemia cells. The results indicated that KYN significantly increased proliferation, transformation, and relapse in leukemia cells, and these effects were suppressed by IDO inhibition.

In summary, this study demonstrated that AHR–IDO–KYN signaling promotes IP-induced oncogenesis in BCP-ALL cells. The strong clinical correlation between the levels of AHR, IDOs, and KYN in patients with ALL underscores the importance of AHR–KYN signaling in BCP-ALL progression. This pathway might represent a new target for the prevention and treatment of BCP-ALL.

## Supplementary Information

Below is the link to the electronic supplementary material.

Supplementary file1 (PDF 93 kb)

## Data Availability

The datasets during and/or analyzed during the current study are available from the corresponding author on reasonable request. Some data may not be made available because of privacy or ethical restrictions.
