# Supplementary material for: Aryl hydrocarbon receptor–kynurenine axis promotes oncogenic activity in BCP-ALL
Source: Cell Biol Toxicol. 2022 Jun 10;39(4):1471–87. doi: 10.1007/s10565-022-09734-0 (PMC10425300; doi:10.1007/s10565-022-09734-0)
Supplement: Supplementary file 1 — Supplementary file1 (PDF 93 kb) [file 10565_2022_9734_MOESM1_ESM.pdf]

Figure S1.

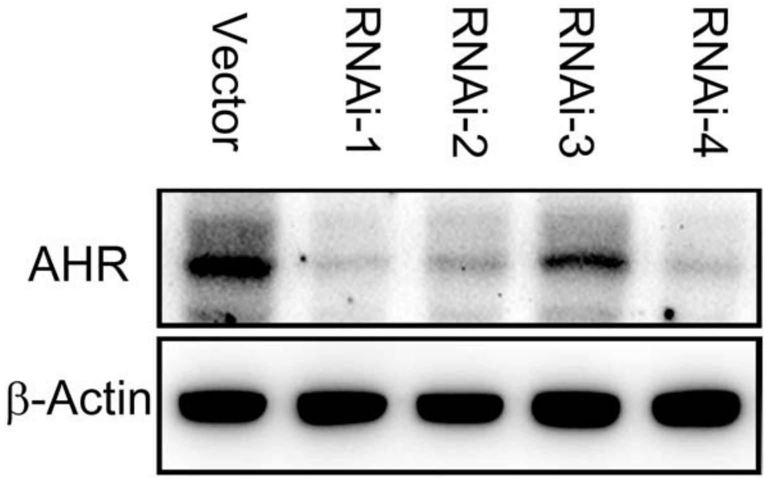

Supplementary Figure 1. Endogenous AHR was silenced in Nalm-6 cells using four difference short hairpin RNA interference.
